# ASSOCIATIONS OF PHYSICAL ACTIVITY ENGAGEMENT WITH CEREBRAL AB AND TAU PET FROM MIDLIFE

**DOI:** 10.1093/geroni/igae098.3149

**Published:** 2024-12-31

**Authors:** Mitzi Gonzales, Daniel Kojis, Nicole Spartano, Emma Thibault, Charles DeCarli, Keith Johnson, Alexa Beiser, Sudha Seshadri

**Affiliations:** Cedars-Sinai Medical Center, Los Angeles, California, United States; Boston University, Boston, Massachusetts, United States; Boston University School of Medicine, Boston, Massachusetts, United States; Massachusetts General Hospital, Boston, Massachusetts, United States; University of California Davis, Davis, California, United States; Massachusetts General Hospital, Boston, Massachusetts, United States; Boston University, Boston, Massachusetts, United States; The University of Texas Health San Antonio, San Antonio, Texas, United States

## Abstract

Higher midlife physical activity engagement has been associated with lower dementia risk in late life. However, the underlying mechanisms contributing to the protective effect remain unclear. The goal of the current study was to evaluate the associations of physical activity with cerebral amyloid beta (Aβ) and tau in a predominately middle-aged community-based cohort, as well as to explore whether the associations differ by age or sex. Participants from the Framingham Heart Study underwent 11C-Pittsburgh Compound B amyloid and 18F-Flortaucipir tau positron emission tomography (PET) imaging. Total physical activity levels were evaluated by self-report using the Physical Activity Index (PAI). Cross-sectional associations between total PAI with regional Aβ and tau PET retention were evaluated using linear regression models adjusted for demographic and cardiovascular risk factors. Interactions with sex and age group were examined and stratified analyses were performed when significant. The sample included 354 participants (mean age 53±8 years, 51% female). Higher total PAI scores were associated with lower entorhinal cortex (β(SE)=-0.021(0.008), p=0.013), inferior temporal lobe (β(SE)=-0.017(0.008), p=0.040), and rhinal cortex (β(SE)=-0.018(0.008), p=0.030) tau PET binding. There were significant interactions with sex with only males displaying inverse associations between total PAI with entorhinal cortex, inferior temporal lobe. and rhinal cortex tau. The results suggest that higher midlife physical activity may confer resistance against tau pathology in regions susceptible to early neuropathological changes. However, the effects may vary based on sex, highlighting the importance of better understanding and tailoring lifestyle interventions to address sex disparities.

